# Correlation Between the Hepatic Expression of Human MicroRNA hsa-miR-125a-5p and the Progression of Fibrosis in Patients With Overt and Occult HBV Infection

**DOI:** 10.3389/fimmu.2018.01334

**Published:** 2018-06-13

**Authors:** Nicola Coppola, Lorenzo Onorato, Marta Panella, Giorgio de Stefano, Nicola Mosca, Carmine Minichini, Vincenzo Messina, Nicoletta Potenza, Mario Starace, Loredana Alessio, Nunzia Farella, Evangelista Sagnelli, Aniello Russo

**Affiliations:** ^1^Department of Mental Health and Public Medicine, University of Campania, Luigi Vanvitelli, Naples, Italy; ^2^Infectious Diseases Unit, AORN Sant’Anna e San Sebastiano, Caserta, Italy; ^3^Department of Environmental, Biological and Pharmaceutical Sciences and Technologies, University of Campania, Luigi Vanvitelli, Caserta, Italy; ^4^IX Interventional Ultrasound Unit for Infectious Diseases, AORN dei Colli, P.O. Cotugno, Naples, Italy

**Keywords:** HBV infection, microRNA, chronic hepatitis, liver fibrosis, occult B infection

## Abstract

**Aims:**

To evaluate the correlation between the hepatic expression pattern of hsa-miR-125a-5p and HBV-DNA and the progression of fibrosis in patients with overt or occult HBV infection.

**Methods:**

We enrolled all the HBsAg-positive treatment naive patients (overt HBV group) and all the HBsAg-negative patients with hepatocellular carcinoma and with a positive HBV-DNA in their hepatic tissue (occult HBV group), who underwent a diagnostic liver biopsy between April 2007 and April 2015. Tissue concentrations of HBV-DNA and hsa-miR-125a-5p were then analyzed by real-time quantitative PCR. Necroinflammatory activity and fibrosis were evaluated according to the Ishak score.

**Results:**

During the study period, we enrolled 64 patients with overt and 10 patients with occult HBV infection. In the overt HBV group, 35 of 64 (54.7%) showed a mild fibrosis (staging 0–2), 17 (26.6%) a moderate fibrosis (staging 3–4), while the remaining 12 (18.7%) had a cirrhosis. All patients in the occult HBV group were cirrhotic. Patients with more advanced fibrosis stage showed a higher mean age when compared with those with mild (*p* < 0.00001) or moderate fibrosis (*p* < 0.00001) and were more frequently male than patients with staging 0–2 (*p* = 0.04). Similarly, patients with occult B infection were older than HBsAg-positive patients. Liver concentrations of miR-125a-5p were significantly higher in patients with cirrhosis (9.75 ± 4.42 AU) when compared with patients with mild (1.39 ± 0.94, *p* = 0.0002) or moderate fibrosis (2.43 ± 2.18, *p* = 0.0006) and were moderately higher in occult than in overt HBV infection (*p* = 0.09). Moreover, we found an inverse correlation, although not statistically significant, between the tissue HBV-DNA levels and the staging of fibrosis.

**Conclusion:**

This study suggests a correlation between the tissue expression of hsa-miR-125a-5p and the progression of liver damage in a group of patients with occult or overt HBV infection. If confirmed, these data suggest the hsa-miR-125a-5p may be a novel biomarker of hepatic damage.

## Introduction

MicroRNAs (miRNAs) are small non-coding RNAs that regulate gene expression at post-transcriptional level by inducing the degradation of target mRNAs or inhibiting their translation in protein (1). They are involved in a large variety of physiological processes playing crucial roles in cell differentiation and development (2). In addition, several studies indicate that miRNAs are important regulators of virus–host interactions (3–5).

HBV represents a leading cause of cirrhosis and hepatocellular carcinoma (HCC) all over the world. World Health Organization estimates that 257 million persons, or 3.5% of the world population, were living in 2015 with chronic HBV infection, which was responsible of more than 900,000 deaths each year (6). The severity of chronic hepatitis B (CHB) is variable, with a clinical presentation ranging from a healthy HBV carriage to the more severe expressions of the disease and with a clinical course ranging from a benign indolent progression over decades to a rapid evolution to liver cirrhosis and HCC (7, 8). Moreover, after HBsAg seroclearance, HBV-DNA can persist inside the hepatocytes, causing a condition known as occult B infection, characterized by HBsAg negativity but persistence of HBV DNA in the liver. The occult HBV infection may be associated with the progression of liver damage and the development of HCC in patients with liver diseases due to different etiologies (9, 10).

Despite the efforts of the scientific community, the interactions between the virus and the host and the mechanisms at the base of liver damage are still largely elusive, which represents a significant barrier to the treatment and the eradication of HBV infection. At this regard, many research groups have investigated the relationship between the expression profile of several miRNAs and HBV replication (11). In particular, it has been shown that hsa-miR-125a-5p, a miRNA expressed in the human liver (12), is able to target a viral sequence within the overlapping polymerase and surface antigen coding regions (13), inhibiting the expression of HBsAg *in vitro*. Moreover, miRNAs are involved in the progression of liver fibrosis at multiple levels, by regulating the activation of hepatic stellate cells, the production of TGF-β, and the expression of matrix metalloproteinases (14).

In this study, we aimed to correlate the hepatic expression pattern of hsa-miR-125a-5p with the concentrations of HBV-DNA in liver tissue and the progression of fibrosis in patients with overt or occult HBV infection.

## Patients and Methods

### Patients

We enrolled in this cross-sectional study all the consecutive HBsAg-positive patients who underwent a diagnostic liver biopsy (overt HBV group) between April 2012 and April 2015 in one of the three liver units participating in the study. The three units involved, two in Naples and one in Caserta, have cooperated in several investigations with the same clinical approach and the same laboratory methods (15, 16). Moreover, of the 68 HBsAg-negative patients with HCC who underwent liver biopsy in the same period, those with a positive HBV-DNA in non-HCC liver tissue were enrolled (occult HBV group).

Exclusion criteria for HBsAg-positive patients (overt HBV group) were HIV, HCV, or HDV coinfection and previous treatment with nucleos(t)ide analogs and/or interferon therapy. For HBsAg-negative patients (occult HBV group), HIV coinfection and previous treatment with interferon-free regimens were regarded as exclusion criteria.

We collected at the enrollment the demographic characteristics of each subject (age, gender, geographical origin). All patients underwent complete physical examination, full liver function tests, assessment of triglycerides, cholesterol, blood cell counts, α-fetoprotein, viral markers (HBV, HCV, hepatitis delta virus-HDV, human immunodeficiency virus-HIV), and liver ultrasound scan.

### Tissue Specimen Collection and Histological Analysis

Liver biopsy was performed for all patients and was advised by the physicians in care, and informed consent was signed by the patient. Liver specimens were fixed in formalin, embedded in paraffin and stained with Masson’s trichrome stain. Liver biopsies were examined by a pathologist who, unaware of the clinical and laboratory data, used the Ishak scoring system to grade the fibrosis (17).

For each patient, a fragments of nearly 3 mg were cut away from the two extremities of the liver biopsies not useful for diagnosis (18) and stored at −80°C in RNAlater solution (Qiagen GmbH, Hilden, Germany) for subsequent molecular analyses. In addition, plasma sample was collected for each patient and stored at −80°C the same day the liver biopsies were performed.

### Ethics Statement

All procedures applied in the study were in accordance with the international guidelines, with the standards on human experimentation of the Ethics Committee of the Azienda Ospedaliera Universitaria-Università della Campania and with the Helsinki Declaration of 1975 and revised in 1983. The Ethics Committee of the Azienda Ospedaliera Universitaria of the University of Campania approved the study (no. 214/2012 and no. 349/2013). All patients signed their informed consent for liver biopsy, the collection and storage of biological samples, and for the anonymous use of their data for research purposes.

### Serological Analysis

HBV and HDV serum markers were sought using commercial immunoenzymatic assays (Abbott Laboratories, North Chicago, IL, USA, for HBsAg, anti-HBs, and anti-HBc, and DiaSorin, Saluggia, VC, Italy, for anti-HDV). The anti-HCV antibody was sought using a third generation commercial immunoenzymatic assay (Ortho Diagnostic Systems, Neckargemund, Germany). Antibodies to HIV 1 and 2 were sought using a commercial ELISA (Abbott Lab., North Chicago, IL, USA). Liver function tests were performed by the routine methods.

### Quantitation of HBV DNA and miR-125a in Tissue Samples

The liver tissues stored at −80°C in RNAlater solution were homogenized by TissueLyser. For all patients in overt and occult HBV groups, the DNA extracted from liver homogenates was analyzed for the presence of the HBV genome by performing a real-time PCR with a wide range of linearity in a Light-cycler 1.5 (Roche Diagnostics, Branchburg, NJ, USA). An external standard curve was made to quantify the HBV genomes present in the samples; the standard was a PCR product cloned with the TA cloning system (Invitrogen k2000-01, Invitrogen, Carlsbad, CA, USA), and 8 µL of the appropriately diluted plasmid was used to generate the standard curve; by this method, the detection limit in plasma samples is estimated at around 40 IU/mL (18, 19). HBV DNA in the liver tissue was quantified in relation to β-globin DNA (LightCycler Control kit Fast star DNA Master Hyprobe, Roche Diagnostics, Branchburg, NJ, USA), a DNA present in all human cells and thus used as a positive control, using LightCycler quantification software (Roche Diagnostics, Branchburg, NJ, USA). The results were expressed as a number of IU/hepatic cell (18, 19).

For the patients in all groups, total RNA was extracted by mirVana™ miRNA isolation kit from liver tissues homogenized by TissueLyser; RT-PCR tests for hsa-miR-125a-5p and RNU6B (used as a reference gene) were carried out using TaqMan miRNA assays from Applied Biosystems.

### Statistical Analysis

Continuous variables were summarized as median and interquartile range, and categorical variables as absolute and relative frequencies. For continuous variables, the differences were evaluated by Wilcoxon rank-sum test; categorical variables were compared by chi-square test, using exact procedures if needed. Odds ratios, with 95% confidence intervals, were estimated by a logistic regression model for evaluating the relationship between age, plasma HBV DNA, liver hsa-miR-125a-5p, and presence of cirrhosis. A *p* value <0.05 was considered to be statistically significant.

## Results

Sixty-four overt and 10 occult HBV patients were included in the study; in the overt group, the median age was 44 (IQR: 14.5) years and 75% of patients were males (Table 1); 56 (87.5) were Italian, 3 (4.7) patients came from Eastern Europe, and the remaining 5 (7.8) from Sub-Saharan Africa. The median ALT level was 0.84 (IQR: 1.18) × upper limit of normal (ULN) and the Prothrombin activity was 90% (IQR: 12.48). All patients had a positive plasma HBV-DNA, with a median viral load of 4.7E + 3 (IQR: 5.2E + 4) IU/mL. 10 of 64 (15.6%) showed a staging 0, 17 (26.6%) a staging 1, 8 (12.5%) a staging 2, 16 (25%) a staging 3, 1 (1.6%) a staging 4, while the remaining 12 (18.7%) had a cirrhosis (staging 6); all but one of them had a compensated liver disease. In the occult group, the median age was 67.5 (IQR: 15.5) years and 8 of 10 patients were males. The median ALT level was 1.17 (IQR: 1.48) × ULN and the Prothrombin activity was 86% (IQR: 8.25). Only three subjects had a positive plasma HBV-DNA, with a viral load ranging from 24 to 811 IU/mL. All patients had an HCV-related cirrhosis, with a decompensated disease (Child-Pugh score B) in 6 of 10 patients.

**Table 1:** Demographic, biochemical, virological, and histological characteristics of the enrolled patients according to the etiologic group.

	Overt HBV group	Occult HBV group
No. patients	64	10
Median age (IQR)	44 (20.5)	67.5 (15.5)
Males, no. (%)	48 (75)	8 (80.0)
Geographical origin, no. (%)		
• Italy	56 (87.5)	10 (100)
• Western Europe	3 (4.7)	0 (0.0)
• Sub-Saharan Africa	5 (7.8)	0 (0.0)
AST/ULN (median, IQR)	0.7 (0.6)	1.35 (1.45)
ALT/ULN (median, IQR)	0.84 (1.18)	1.17 (1.48)
PT% (median, IQR)	90 (12.48)	86 (8.25)
HBV DNA positivity, no. (%)	64 (100)	3 (30)
Plasma HBV-DNA load, IU/mL (median, IQR)	4.7E + 3 (5.2E + 4)	24–811^a^
Fibrosis score (Ishak), no. (%) of patients with
• 0	10 (15.6)	0 (0)
• 1	17 (26.6)	0 (0)
• 2	8 (12.5)	0 (0)
• 3	16 (25.0)	0 (0)
• 4	1 (1.6)	0 (0)
• 6	12 (18.7)	10 (100)
- Child-Pugh score A no. (%)	11 (17.2)	4 (40)
- Child-Pugh score B no. (%)	1 (1.6)	6 (60)

*^a^Range*.

*IQR, interquartile range; ULN, upper limit of normal*.

All patients were HIVAb and HDVAb negative; none of the patients in the overt HBV group was HCV-coinfected; two of them were HBeAg positive. Patients with more advanced fibrosis stage showed a higher median age when compared with those with mild (68.5, IQR: 2 vs 40, IQR: 15.5, *p* < 0.00001) or moderate fibrosis (40, IQR: 14, *p* < 0.00001) and were more frequently male than patients with staging 0–2 (91.7 vs 60.0%, *p* = 0.04). Similarly, patients with occult B infection were older than HBsAg-positive patients (67.5, IQR: 15.5 vs 44, IQR: 14.5, *p* = 0.00017). A lower median ALT level was found in patients with mild when compared with patients with moderate fibrosis (0.8, IQR: 0.96 vs 1.24, IQR: 1.8, *p* = 0.02); furthermore, a higher prothrombin activity was found in patients with mild fibrosis when compared with cirrhotic subjects (93, IQR: 12.5 vs 85, IQR: 9.4, *p* = 0.02). Finally, we found a higher concentration of HBV-DNA both in plasma (1.37E + 4 IU, IQR: 9.9E + 5 vs 3.6E + 2 IU, IQR: 1.16E + 4, *p* = 0.03) and in liver tissue (0.14 IU/cell, IQR: 0.27 vs 0.007 IU/cell, IQR: 0.046, *p* = 0.01) when we compared patients with moderate fibrosis to cirrhotic patients.

**Figure 1:**
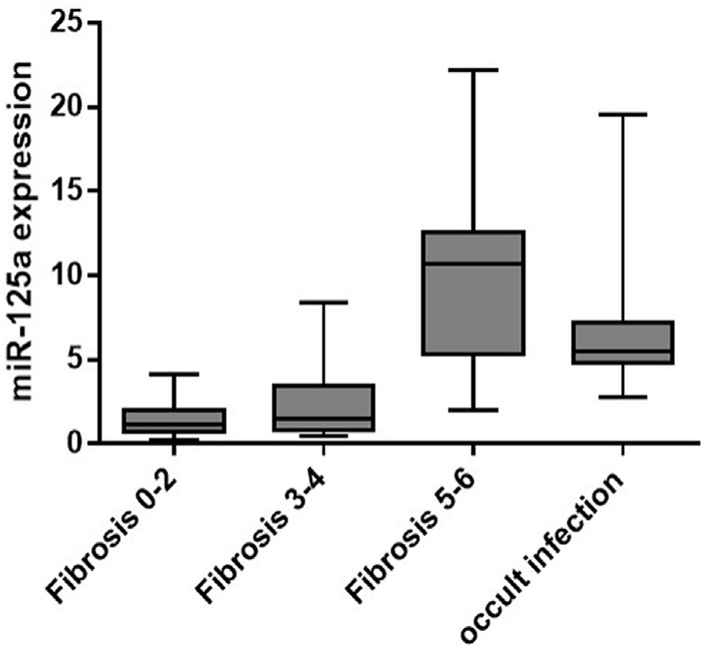
miR-125a-5p expression according to liver fibrosis.

In overt HBV group, liver concentrations of miR-125a-5p (Table 2; Figure 1) were significantly higher in patients with fibrosis score of 5 or 6 (cirrhosis; median, IQR: 10.7, 6.9 AU) when compared with patients with mild (fibrosis score 0–2; median, IQR: 1.13, 1.04 AU, *p* < 0.00001) or moderate fibrosis (fibrosis score 3 or 4; median, IQR: 1.53, 2.55 AU, *p* = 0.0002). The 10 patients in occult HBV group, all cirrhotic, showed a high liver concentrations of miR-125a-5p (median, IQR: 5.47, 2.43 AU). Patients with higher liver HBV-DNA concentrations showed a slightly lower miRNA expression (*p* = 0.24 including all patients; *p* = 0.35 excluding the occult HBV group); similarly, no correlation was found between the miRNA expression and the plasmatic viral load (*p* = 0.11 including all patients; *p* = 0.13 including only the HBsAg-positive patients, data not shown).

**Table 2:** Characteristics of HBV patients stratified according to liver fibrosis.

	Overt HBV group			Occult HBV group	*p* Value			
	Staging 0–2	Staging 3–4	Staging 5–6		Staging 0–2 vs 3–4	Staging 0–2 vs 5–6	Staging 3–4 vs 5–6	Occult vs overt HBV group
No. patients	35	17	12	10				
Age (median, IQR)	40 (15.5)	40 (14)	68.5 (2)	67.5 (15.5)	0.36	**<0.00001**	**<0.00001**	**0.00017**
Males, no. (%)	21 (60)	14 (82.3)	11 (91.7)	8 (80.0)	0.11	**0.04**	0.47	0.73
AST/ULN (median, IQR)	0.7 (0.57)	0.76 (1.05)	0.7 (1)	1.35 (1.46)	0.09	0.38	0.28	**0.01**
ALT/ULN (median, IQR)	0.8 (0.96)	1.24 (1.8)	0.5 (1.31)	1.18 (1.47)	**0.02**	0.32	0.08	0.14
PT% (median, IQR)	93 (12.5)	91.5 (13.3)	85 (9.4)	86 (8.25)	0.29	**0.02**	0.05	0.22
Plasma HBV-DNA UI/ml (median, IQR)	4.7E + 3 (4.7E + 4)	1.37E + 4 (9.9E + 5)	3.6E + 2 (1.16E + 4)	//	0.18	0.16	**0.03**	**//**
Liver HBV-DNA IU/cell (median, IQR)	0.03 (0.1)	0.14 (0.27)	0.007 (0.046)	0.015 (0.34)	0.14	**0.04**	**0.01**	0.37
miR-125a-5p AU (median, IQR)	1.13 (1.04)	1.53 (2.55)	10.7 (6.9)	5.47 (2.43)	0.11	**<0.00001**	**0.0002**	**0.006**

*IQR, interquartile range; ULN, upper limit of normal; cp, copies; AU, arbitrary units*.

*P values <0.05 are displayed in bold font*.

To confirm the association between liver hsa-miR-125a-5p and the presence of an advanced liver disease and to avoid a possible confounding effect of other factors such as age and the plasma HBV-DNA level, a multivariate logistic regression analysis was performed. Multivariate analysis identified liver hsa-miR-125a-5p as an independent predictor of fibrosis >5 (OR: 2.08, CI 95%: 1.11–3.85, *p* = 0.02) (Table 3).

**Table 3:** Multiple logistic regression analysis for independent predictors of staging >5.

	OR	95% CI		*p* Value
		Lower limit	Upper limit	
Age	1.37	0.94	1.96	0.10
miR-125a-5p	2.08	1.11	3.85	**0.02**
Plasma HBV-DNA	0.49	0.16	1.51	0.21

*OR, odds ratio; CI, confidence interval*.

*P values <0.05 are displayed in bold font*.

## Discussion

HBV represents one of the most important issues for global health. The spectrum of clinical conditions caused by HBV infection is wide, ranging from asymptomatic carriers to liver cirrhosis and hepatocellular cancer, and several authors have tried to investigate the pathogenesis of liver damage induced by this virus.

It is well known that miRNAs can regulate almost every biological process in all cell types, including hepatocytes and other liver cells. Not surprisingly, the role of miRNAs in liver fibrogenesis and carcinogenesis has emerged in the recent years as one of the most interesting issue in the field of hepatology. Many studies have highlighted the expression pattern of several miRNAs, both in plasma and in liver, in patients with chronic HBV infection (20, 21). In a recently published paper, Singh and coworkers (22) investigated the expression profile of several miRNAs by microarrays in liver biopsy samples from 65 patients with different stages of HBV infection and 7 healthy controls. They found in immune tolerant patients higher levels of miR-199a-5p, miR-221-3p, and Let-7a-3p involved in the regulation of innate immune response. Conversely, in the advanced fibrosis group they demonstrated an upregulation of miR-1 and miR-10b-5p, and a downregulation of miR-20b-5p and miR-455-3p, implicated in immune response and cellular senescence.

miR-125a-5p is present in all animals with bilateral symmetry; in mammalians, it is expressed in most tissues (23) where negatively regulates cell proliferation (24). Indeed, it is downregulated in several types of tumors (25, 26), including hepatocellular cancer (27, 28). However, its role in liver is not limited to the tumor suppressor activity; a few studies have investigated its role in liver fibrosis progression. In 2012, Park et al. (29) showed that TGF-β, a profibrogenic cytokine, could induce an upregulation of hsa-miR-125a-5p in HBV transfected hepatocytes. A more recent study by Li et al. (30) demonstrated an upregulation of this miRNA in a murine model of carbon tetrachloride-induced liver fibrosis; furthermore, they showed how the downregulation of miR-125a-5p could prevent the activation of hepatic stellate cells *in vitro*. The same group demonstrated a strong correlation between serum concentrations of miR-125a-5p and staging of fibrosis in 91 patients with CHB (31). They also found a significant positive correlation between the expression levels of the miRNA and the serum HBV-DNA.

In a previous study, we analyzed a little cohort of 27 HBsAg/anti-HBe-positive patients and correlated the liver concentrations of miR-125a-5p with the clinical, virological, and histological characteristics of the enrolled patients (19, 32). Liver miRNA expression was identified as an independent predictor of higher necroinflammatory activity (HAI > 6) and more advanced liver fibrosis (staging > 2). Furthermore, more elevated miRNA expression was found in patients with higher serum and liver HBV-DNA levels. In this study, on a larger HBV population, 64 consecutive patients with overt and 10 with occult HBV infection were enrolled and the expression levels of liver hsa-miR-125a-5p were determined, along with clinical, biochemical, and histological parameters. As expected, patients with more advanced liver disease were older than patients with mild or moderate fibrosis. Furthermore, they were more frequently male; these data can be explained with a higher incidence in male gender of HCC, which represents one of the main indications for liver biopsy in cirrhotic subjects. Finally, as expected, patients with initial fibrosis (staging 0–2) showed a higher prothrombin activity than cirrhotic subjects and a lower ALT level than patients with more advanced liver damage (staging 3–4). Regarding the virological characteristics, we found a lower plasma and liver viral load in cirrhotic subjects than in patients with less advanced disease; this could be related to the clinical and demographic features of patients with mild or moderate fibrosis (i.e., younger and more frequently HBeAg-positive patients).

In the HBV-infected patients, we demonstrated a significant correlation between the miRNA concentrations and the progression of liver fibrosis. In fact, the liver has-miR-125a-5p concentrations were higher in the patients with higher fibrosis score. However, we did not find a correlation with the plasma and tissue HBV viral load. This is may be due to the inclusion in this study of patients with advanced stages of fibrosis, not included in our previous study, infection by different HBV strains inducing a lower miR-125a expression response (all patients in the former study were infected with a genotype D, while five patients in this paper have a genotype E and two a genotype A), or different serological characteristics (e.g., HBeAg-positive patients, not included previously).

The study has some limitations. First, the cross-sectional design does not allow to clearly evaluate the impact of single factors on the progression of liver damage, as a prospective study could do. Moreover, the lack of control groups with different etiologies and virological characteristics makes it difficult to understand the real correlation between the miR-125a expression and the liver fibrosis. However, the multivariate analysis that identified the miRNA concentrations as independent predictor of advanced liver damage (regardless of the viral load) suggest a role of miR-125a-5p in the process of fibrogenesis, not correlated to the inhibitory effect on HBV replication.

## Conclusion

This study demonstrates a correlation between the tissue expression of hsa-miR-125a-5p and the progression of liver damage in a group of patients with occult or overt HBV infection. However, further studies are needed to investigate the role of this miRNA in pathogenesis of HBV infection, to assess novel biomarkers of hepatic damage. Moreover, we need more data to understand the role of this miRNA in the process of fibrogenesis induced by etiologies other than HBV.

## Ethics Statement

All procedures applied in the study were in accordance with the international guidelines, with the standards on human experimentation of the Ethics Committee of the Azienda Ospedaliera Universitaria-Università della Campania and with the Helsinki Declaration of 1975 and revised in 1983. The Ethics Committee of the Azienda Ospedaliera Universitaria of the University of Campania approved the study (no. 214/2012 and no. 349/2013). All patients signed their informed consent for liver biopsy, the collection and storage of biological samples, and for the anonymous use of their data for research purposes.

## Author Contributions

Concept and design: NC, AR, and ES. Production and analysis of data: MS, CM, MP, NM, and NP. Patients inclusion, collection of samples, analysis of data: LO, GS, NF, LA, and VM. Writing of article: NC, LO, and AR.

## Conflict of Interest Statement

The authors declare that the research was conducted in the absence of any commercial or financial relationships that could be construed as a potential conflict of interest.
